# Do *APOE4*, malnutrition, and long COVID-19 compound the risk factors for stroke in adverse environments?

**DOI:** 10.3389/fnut.2024.1422218

**Published:** 2024-07-23

**Authors:** Alexander Vasconcelos Buzaglo, Carlos Meton de Alencar Gadelha Vieira, Gabriella Cunha Vieira Ciurleo, Ludmila Belayev, Reinaldo B. Oriá

**Affiliations:** ^1^Laboratory of the Biology of Tissue Healing, Ontogeny and Nutrition, Department of Morphology and Institute of Biomedicine, School of Medicine, Federal University of Ceara, Fortaleza, Ceará, Brazil; ^2^School of Medicine, Neuroscience Center of Excellence, Louisiana State University Health Sciences Center, New Orleans, LA, United States

**Keywords:** APOE, malnutrition, long COVID, stroke, adverse environment

## *APOE4* under adverse environments

Apolipoprotein E (apoE) is a 299 amino acid protein with vital functions in transporting plasma cholesterol from peripheral tissues to the liver to be metabolized (1). The human apolipoprotein E gene (*APOE)* is polymorphic, carrying three common alleles that encode distinct apoE isoforms (apoE4, apoE3, and apoE2), displaying differing biological functions and binding affinities to LDL receptors. ApoE4 is primarily expressed in the liver, functioning as a ligand during the receptor-mediated endocytosis of lipoprotein particles (2, 3), resulting in increased total cholesterol and LDL levels, whereas apoE2 has opposite effects (4). ApoE4 has immunomodulatory roles in different experimental models (5, 6) and may have distinct effects under adverse and privileged environments. In the CNS, apoE is the primary apolipoprotein regulating lipid metabolism, being mainly produced by astrocytes. ApoE is involved in brain cholesterol recycling and redistribution, affecting membrane maintenance, organelle biogenesis, and synaptogenesis, which are essential for neuroplasticity (7). ApoE4 is highly expressed in glial cells and is recognized to bind to brain amyloid plaques and is a culprit of unbalanced neuroinflammatory responses in Alzheimer's disease (AD) patients and declining health status (8). *APOE4* carriers have a significantly increased risk of acquiring late-onset Alzheimer's and cardiovascular diseases (9). Following cerebral focal ischemia, apoE4 was found to exacerbate infarction size and hemiparesis in transgenic mice, as well as increased peri-infarct GFAP-associated reactive astrocytosis when compared to apoE3 and apoE2 (10, 11). Although much is known about how apoE-cholesterol-derived from astrocytes affects the build-up of synaptic circuits (12) and impairment in AD (the latter according to apoE2 < apoE3 < apoE4) (13), a gap of knowledge remains on apoE role on brain synapses following stroke under adverse environments.

Despite the adverse effects of *APOE*4 in modern Western societies, the prevalence of *APOE*4, considered the ancestral *APOE* allele, is higher in hunter-gatherer remote populations (4). A reason for *APOE4* prevalence in populations with less access to health services may be the high exposure to endemic infectious pathogens (14). *APOE4* may behave as an antagonistic pleiotropic gene that may benefit reproductive fitness and better coping against environmental pathogens to improve population growth under famine and high infectious disease rates (1, 15), with a trade-off toward enhanced innate immunity against enteric pathogens for immediate survival and thriving, however with sustained up-regulation of inflammatory responses, which may be harmful for long-term chronic diseases under more privileged environments. Indeed, the *E4* allele is highly prevalent in tropical populations that endure higher parasite burdens in adverse environments. Interestingly, *APOE4* occurrence was associated with less cognitive decline in individuals with eosinophilia, a proxy of helminthic infections (16).

## The double burden of malnutrition crisis and the COVID-19 pandemic

The double burden of malnutrition (DBM) is a phenomenon that may occur when undernutrition and overnutrition afflict individuals during their lifespan or societies with troublesome consequences for health, predisposing individuals to other chronic diseases, such as atherosclerosis, stroke, diabetes, and non-alcoholic liver steatosis (17–19). DBM has been recognized in developing countries with emerging economies, such as Brazil, South Africa, and India, and is expected to be associated with the rise of diabetes in close association with cardiovascular diseases in poverty-troubled settings (20). Recently, the COVID-19 pandemic has exacerbated the global malnutrition crisis along with staggering rates of poverty and food insecurity, undermining essential public health and social programs (17, 21).

The multinational lockdowns and the resulting home confinement of over 2.6 billion people (22) were responsible for a change in lifestyle from outdoor to indoor activities, instigating sedentary lifestyles accompanied by a range of metabolic and endocrine stressors arising from unhealthy eating habits, dysfunctional sleep patterns, smoking, and alcohol intake (23), all of which are important risk factors for the development of atherosclerosis and stroke (11). Stroke has been a public health concern following the COVID-19 pandemic, with a rise in cases and mortality in all age groups, especially those with comorbidities (24).

Post-COVID-19 Syndrome, also known as “long COVID,” is a post-viral syndrome characterized by symptoms persisting after the end of a COVID-19 infection that cannot be better explained by an alternative diagnosis (9, 25), affecting between 32.6% to 87% of patients that underwent hospitalization as well as some non-hospitalized patients (9). It is a multifactorial condition with hundreds of possible symptoms and affects various tissues, organs, and systems (25). According to the US Household Pulse Survey and corroborated by Robertson et al., around 7.3% of the U.S. population above 18 years were experiencing long COVID as of July 2022, and a proportion of 2.8% has been documented in the UK for those above 2 years of age (26). Neuropsychiatric symptoms appear to be related to neuroinflammation and blood-brain barrier (BBB) disruption, caused by exaggerated immune responses to SARS-CoV-2, symptomatically resembling post-viral fatigue syndrome (also known as chronic fatigue syndrome), all of which may be aggravated by malnutrition states (9). Females, the elderly, and people with chronic conditions are at a higher risk of developing long COVID (27).

## The interplay between *APOE4*, COVID-19, and the global stroke crisis

An emerging body of evidence implicates the cumulative burden of lifelong infections, termed Chronic Infectious Burden, to an increased risk of atherosclerosis and stroke, to which both chronic and acute infections are significant contributors. During the COVID-19 pandemic, a rise in stroke cases was recognized, with the highest risk seen during the first 3 days after COVID-19 diagnosis and decreasing over several weeks (28). The etiology for this increased risk is multifaceted, potentially varying between different conditions; however, pre-existing comorbidities, such as aging, obesity, and diabetes, were strong co-drivers (29).

One of the main features of COVID-19 is the advent of a heightened, dysregulated immune response brought about by the virus' modulation of the adrenergic and hypothalamic-pituitary-adrenocortical axis, inducing a hyperactive innate immune response, leading to neutrophilia and elevated cytokine production, directly followed by immunosuppression and lymphopenia, with these effects and the degree of the viral load—and, therefore, the graveness of the prognosis. COVID-19-associated systemic infection biomarkers include IL-6, erythrocyte sedimentation rate (ESR), TNF, IFN-γ, IP-10, MCP-3, and HGF (28, 30). In addition, vascular cell adhesion molecules (VCAMs), plasmatic levels of free DNA, fibrinogen-to-albumin ratio, CRP, PCT, and ferritin are also increased. A decrease in CD3+, CD4+, CD8+ T cells, and NK cells is observed proportionally to disease severity (30).

The COVID-19-associated inflammatory storm has been linked with an increased risk of ischemic stroke through the formation of thrombi as a by-product of pro-coagulant and peripheral pro-inflammatory responses, as well as by worsening two stroke-related risk factors: atherosclerosis and atrial fibrillation (31, 32). Atherosclerosis is potentiated through elevated macrophage and T-cell responses, which begin to form a lipid-rich core due to the accumulation of apoptotic cell debris and lipid pools in the vessel wall throughout the local inflammation, followed by the release of destabilizing factors such as IFN-γ and TNF, as well as lytic enzymes such as metalloproteinases, that expose the atheromatous plaque's core and potentiate its rupture and thromboembolism (11, 31). Pneumonia-associated hypoxia that often accompanies COVID-19 may also contribute toward a prothrombotic state (32).

COVID-19 can induce microvascular injury, directly inflicting endothelial cell damage, by a viral protease to cleave the NF-kB essential modulator, ultimately leading to BBB disruption and brain neuroinflammation. BBB damage and subsequent increased capillary permeability may compromise CNS perfusion, potentially amplifying the risk and severity of hypoxic insults, such as those caused by stroke (28). Hospital stroke incidence was 7–8 times higher in COVID-19 than influenza patients (31). COVID-19 may induce a particularly hypercoagulable state via increased D-dimer, fibrinogen, factor VIII, von Willebrand factor, antiphospholipid antibodies, and lupus anticoagulant concentrations (11). Such a cascade of pro-coagulant events is triggered and amplified by a cytokine release storm, endothelial injury, complement system, and neutrophil activation (28). COVID-19-driven platelet-rich thrombosis in alveolar capillaries and small vessels has been evidenced in post-mortem lung tissue (31).

SARS-CoV-2 has been found in thrombi of brain arteries from acute ischemic stroke patients, who also showed systemic high neutrophil-to-lymphocyte ratio as well as higher angiotensin-converting enzyme 2 (ACE2) expression (32), and COVID-19 has also been implicated in causing acute cardiac injury, arrhythmias, and atrial fibrillation, which are commonly found in COVID-19 patients, with increased risk of thromboembolism and subsequent stroke (25, 28).

ACE2 is expressed in many tissues and catalyzes the degradation of angiotensin II into angiotensin 1–7, which contributes to anti-hypertensive effects. Dysfunction of ACE2 expression has been implicated in a variety of pathological conditions, ranging from hypertension, diabetes mellitus, acute lung injury, and Alzheimer's disease (33, 34). ACE2 is a well-recognized gateway for SARS-CoV-2 entry into the host cells—indeed, this likely plays a role in low lymphocyte and leukocyte counts in patients due to the protein's expression on their surface (30). However, APOE4 has been found to downregulate ACE2 protein expression, leading to a dysregulation of the renin-angiotensin system (33). The ACE2 polymorphism rs2285666 was associated with the risk of developing stroke in patients with type 2 diabetes mellitus (34). This finding was corroborated by Liu et al., who showed that another ACE2 polymorphism (rs4240157) correlated with an increased risk of stroke in these patients in a Chinese population (35). Further research is necessary to better elucidate the relationship between APOE and ACE2 polymorphisms associated with COVID-19 outcomes and stroke risk. Interestingly, an *in vitro* study found that incubation with recombinant apoE3 and apoE4 partially inhibited COVID-19 virus' entry into cells stabling expressing human ACE2; however, due to apoE4′s more compacted structure, it was less effective at inhibiting viral entry compared to apoE3 (36).

Long COVID-19 also seems to increase the risk of stroke, particularly in genetically prone individuals, and has been found to negatively impact stroke patients and the health services they rely upon (37), yet long COVID-driven stroke has not been sufficiently investigated amidst a context of an aging-related global rise in cardiovascular diseases that has been taking place for the last 30 years (38). According to a report by the British Heart Foundation, excess deaths resulting from cardiovascular diseases, including cerebrovascular diseases, remained high in the UK even as fatalities associated with COVID-19 have fallen, raising questions about other potential causes, with disruptive healthcare services that may be aggravated by COVID-19 post-pandemic effects (39).

*APOE* alleles were not found to predispose ischemic or hemorrhagic strokes, though the *E2* and *E4* alleles were overrepresented in brain amyloid angiopathy-related hemorrhage (40). *APOE4* was found to affect prognoses, with a small positive effect on ischemic events and strokes more pronounced in homozygotes. Nevertheless, the *E4* allele appears to lower survival rates for hemorrhagic stroke but improve it for ischemic stroke (40, 41). Following ischemic and hemorrhagic insults, an increased expression and neuronal apoE uptake are seen. Furthermore, apoE-deficient mice develop a larger infarct volume following such insults (40), suggesting that apoE plays essential roles in CNS repair depending on the isoform (38, 42). ApoE4 appears to have a less effective anti-inflammatory function, even being a pro-inflammatory factor, when compared to other apoE isoforms (2), aggravating the neurological damage and clinical outcomes in various conditions, such as Alzheimer's disease and stroke.

BBB disruption and vulnerability have been found in APOE4-targeted replacement mice compared to APOE2 and APOE3, leading to postsynaptic interactome dysfunction and behavioral deficit (43). APOE4 occurrence in mice has been associated with an increased reduction in brain pericyte number and coverage surface; such pericyte impairment has been observed in AD (44). Cerebral pericytes display beneficial roles following stroke, with a multifaceted role in angiogenesis and subsequent neurogenesis (45). SARS-CoV-2 may infect brain pericytes, and increased pericyte ACE2 expression has been associated with the severity of neurological symptoms in infected patients (46).

ApoE4 has been found to increase cerebral microhemorrhages in COVID-19 patients, likely through perivascular damage and/or microglial activation. This worsens the infection's outcomes and serves as a risk factor for lasting mental fatigue following severe COVID-19 (47). ApoE4's compact structure and low spatial interference have been found to facilitate the SARS-CoV-2 virus entry into the cell compared to other apoE isoforms. Lastly, the *APOE* single nucleotide polymorphisms rs428358 and rs7412 were also linked to ischemic cerebral infarction (48).

## Conclusion

This opinion paper calls attention to the potential compound effects of DBM and long COVID-19 in *APOE4* carriers to substantially increase the risk for stroke, particularly in adverse and poor settings, where the mitigation of the problem is hampered by difficult access to urgent and specialized healthcare.

## Author contributions

AB: Writing – original draft, Writing – review & editing. CV: Writing – original draft, Writing – review & editing. GC: Writing – original draft, Writing – review & editing. LB: Writing – original draft, Writing – review & editing. RO: Conceptualization, Supervision, Writing – original draft, Writing – review & editing.
